# Stigmatizing attitudes and predictors of empathy toward mentally ill patients among psychiatric and mental health nurses

**DOI:** 10.1186/s12912-025-02926-z

**Published:** 2025-04-24

**Authors:** Warda Elshahat Hamed, Ashjan Saeed Babegi, Hind Abdallah Modawi Elamin, Nesma Ahmed Kamel, Ana Maria Escolano-Castillo, Hamad Ghaleb Dailah, Rania Rabie Eletreby

**Affiliations:** 1 Psychiatric and Mental Health Nursing Department, Faculty of Nursing, Mansoura University, Mansoura, Egypt; 2 College of Nursing and Health Sciences, Jazan University, Jazan, Saudi Arabia; 3 Human physiology, College of Nursing and Health Sciences, Jazan University, Jazan, Saudi Arabia; 4 Nursing Administration and Management, College of Nursing and Health Sciences, Jazan University, Jazan, Saudi Arabia; 5 College of Nursing and Health Sciences, Jazan University, Jazan, Saudi Arabia

**Keywords:** Stigma, Attitude, Empathy, Mentally ill patients

## Abstract

**Background:**

Nurses’ personal experiences and relationships with patients suffering from mental disorders are linked to bias and discrimination. A notable association exists between the desire to work in mental health settings and prejudice, as well as between educational attainment and stereotypical beliefs. Mental health nurses need to possess a strong capacity for empathy and positive attitudes towards patients with mental disorders.

**Aim of the study:**

This study aimed to investigate predictors of empathy and stigmatizing attitudes toward mentally ill patients among psychiatric and mental health nurses.

**Method:**

A descriptive correlational cross-sectional research design was used in this study to assess levels of empathy and stigma and to identify predictors of empathy as well as relationships between variables. A convenience sample of 122 psychiatric and mental health nurses was selected as respondents from two settings: the psychiatric inpatient and outpatient departments of Mansoura University Hospitals and Demera Mental Health Hospital which are situated in Dakahlia Governorate in the Delta area, Egypt.

**Tools:**

Three instruments were used in this study, including a socio-demographic questionnaire, the Opening Mind Scale for Health Care Providers (OMS-HC), and the Perth Empathy Scale (PES).

**Results:**

This study found that nearly three-quarters (70.5%) of nurses held low stigmatizing attitudes toward mental illness, while about one-third (29.5%) displayed high stigmatizing attitudes. Most participants (64.8%) scored moderately on the Perth Empathy Scale. A significant association was identified between higher education in psychiatric nursing, years of experience, and increased stigmatizing attitudes toward mental illness. Additionally, a negative relationship was found between stigmatizing attitudes and empathy, indicating that higher stigma was linked to lower empathy scores. The results also suggest that positive attitudes toward mentally ill patients are associated with higher levels of empathy.

**Conclusion:**

Positive attitudes are linked to higher education, more experience, and greater empathy. Higher education and years of experience, along with a positive attitude predicted higher levels of empathy toward mentally ill patients. Targeted interventions, such as workshops, role-playing, and Cognitive Behavioral Therapy (CBT) programs, are recommended to reduce stigma and enhance empathy among psychiatric nurses.

**Clinical trial number:**

Not applicable.

## Introduction

Globally, more than 20% of the population has experienced at least one form of mental illness in their lifetime [1]. Often, these vulnerable individuals encounter stigmatizing attitudes and discrimination not only from the general public but also from healthcare professionals, in particular, Psychiatric and Mental Health Nurses (PMHNs) [2–5]. Stigma is frequently seen as a “second disease” for individuals with mental illness because these individuals suffer the burden of their illness along with its negative repercussions associated with the public and structural stigma [6, 7]. The Middle East has high and rising rates of mental health disorders, driven by ongoing wars, conflicts, and sociocultural, religious, and economic factors that exacerbate stigma. Community-based studies report prevalence rates ranging from 15.6 to 35.5%, with higher rates in nations facing complex emergencies such as wars and famines. Despite efforts to destigmatize mental health, individuals with mental illnesses in Arab countries often face societal rejection and discrimination [8].

Stigma involves social rejection, marginalization, or devaluation of an individual or group perceived as different or abnormal [9–11]. It consists of three distinct components: stereotypes, prejudice, and discriminatory behaviors [12]. Stereotypes are cognitive frameworks reflecting prevailing ideas and negative beliefs regarding the characteristics of a specific group [12]. If individuals embrace these stereotypes, they may experience adverse emotional responses, demonstrating social prejudice that leads to discrimination and isolation of the patient [12]. Consequently, individuals with mental illness find themselves in an unfavorable social predicament.

Many people perceive individuals with mental illness as dangerous or incompetent, preferring to maintain social distance and avoid interacting with them [7, 13]. As a result, this stigmatized group becomes subjected to ostracization and discrimination at both individual and systemic levels [7]. The stigma associated with mental illness leads to various challenges, including difficulties in acquiring housing, finding employment, and accessing legal and healthcare services [14]. These challenges result in social isolation [14], feelings of shame [15], poor self-esteem [11], discontentment with life, and mental health issues [16]. Stigmatizing views detrimentally affect recovery progress, the pursuit and acceptance of psychotherapy [16], and compliance with pharmacological treatments [17].

Stigma impedes attempts to prevent, treat, and recover from mental illness. Health professionals’ perceptions of schizophrenia, depression, and substance abuse are consistent with the general public. Nursing staff often believe that people with mental illnesses are dangerous, unpredictable, and emotionally unstable. They may feel worry, guilt, and hostility toward patients with psychiatric illnesses. Due to the traditional impression of danger, healthcare staff are less willing to provide care to these patients autonomously, imposing additional constraints on recovery rates and the quality of care offered [3, 18].

Empathy, the capacity to comprehend and participate in the emotions of others, is crucial for psychiatric nurses. It enables them to understand and appreciate the significance of patients’ emotions and thoughts, fostering a trusting environment and promoting favorable health outcomes such as decreased distress, anxiety, and depression [19]. Empathy helps nurses retain impartiality, prioritize patient-centred communication, and avoid emotional exhaustion and inappropriate emotional attachment [20]. Conversely, a lack of empathy can result in a failure to provide necessary information and emotional support, leading to heightened distress, anxiety, and ineffective coping mechanisms for patients [21].

Research has shown that factors such as duration of employment, exposure to stressful situations, limited time, limited professional networking opportunities, and strain on the healthcare system contribute to challenges in fostering empathetic interactions among healthcare professionals. These factors can lead to a decline in empathy and an increase in burnout, with 30–40% of nurses globally reporting symptoms of burnout, characterized by depersonalization, emotional weariness, and a lack of personal accomplishment. Elevated levels of empathy serve as a safeguard against burnout [22]. Other factors such as educational level, skills, expertise, socio-demographic, cultural, and religious characteristics can also affect the empathetic relationship between psychiatric nurses and patients. Nurses’ personal experiences and relationships with patients suffering from mental disorders are linked to bias and discrimination. A notable association exists between the desire to work in mental health settings and prejudice, as well as between educational attainment and stereotyped beliefs [23, 24]. In the Middle East, where mental health services are underdeveloped and often culturally constrained, understanding the interplay between stigma and empathy among psychiatric nurses is essential [25] Empirical evidence suggests that stigma reduction and empathy training can significantly enhance the quality of mental health care in these settings [26].

Mental health nurses need to possess a strong capacity for empathy and maintain positive attitudes. The current research study investigated stigmatizing attitudes toward mentally ill patients in Egypt among psychiatric and mental health nurses [9, 27, 28]. In Egypt, mental health stigma presents significant barriers to treatment and recovery for individuals with mental illnesses. The pervasive stigmatization of these patients is rooted in cultural norms that emphasize secrecy about mental health issues, often associating them with weakness or shame [9]. These stigmas are reinforced by societal beliefs and religious interpretations, which sometimes frame mental illness as a result of spiritual or moral failings, further isolating those affected [29, 30]. Despite these challenges, mental health literacy in Egypt remains limited, contributing to poor awareness and delayed help-seeking behavior. Cultural expectations often prevent individuals and families from openly discussing mental health issues, exacerbating stigma at the community level [29]. This underscores the importance of fostering empathy and reducing stigma among mental health professionals, especially nurses, to improve care quality and patient outcomes. By exploring the predictors of empathy and examining stigmatizing attitudes, this study contributes to the understanding of how these factors interplay within the unique cultural and healthcare context of Egypt. Addressing these issues is critical for designing interventions that not only enhance empathy but also alleviate stigma, ultimately promoting equitable and compassionate care for individuals with mental illnesses. However, there is a dearth of research examining both stigma and empathy among Egyptian nurses, in particular PMHNs. Therefore, the purpose of this study is to investigate predictors of empathy and stigmatizing attitudes toward mentally ill patients among psychiatric and mental health nurses (PMHNs). Specifically, this study aims to: (1) Evaluate the extent of stigmatization and empathy toward patients with mental disorders among psychiatric mental health nurses (PMHNs). (2) Investigate the correlation between stigmatizing attitudes toward patients with mental disorders, sociodemographic characteristics of their primary care nurses, and their empathizing attitude. (3) Investigate the predictors of empathy among PMHNs, with a particular emphasis on socio-demographic variables and stigmatizing attitudes toward patients with mental disorders.

### Study’s hypotheses

#### H1

The nurses have a high stigmatizing attitude toward mentally ill patients.

#### H2

The stigmatizing attitudes of the nurses have a significant correlation with their sociodemographic characteristics and level of empathy.

#### H3

Sociodemographic characteristics and stigmatizing attitudes are predictors of empathy of mental health nurses toward their patients.

## Method

### Study design and settings

A correlational descriptive cross-sectional research design was used in this study to assess levels of empathy and stigma and to identify predictors of empathy as well as relationships between variables [31]. The study was conducted in two settings: the psychiatric inpatient and outpatient departments of Mansoura University Hospitals and Demera Mental Health Hospital both situated in the Dakahlia Governorate in the Delta area region of Egypt. These settings offer psychiatric care for individuals of all ages facing psychotic, neurotic, and substance use disorders.

### Sample and sampling procedure

A convenience sample of 122 psychiatric and mental health nurses working in these settings was enrolled based on their appropriateness and willingness to participate in the study. Additionally, a snowball sampling strategy was employed to enhance recruitment and ensure a more representative sample of the population.

The sample size was calculated using the following formula Based on data from the literature [12], to calculate the sample size with precision/absolute error of 5% and type 1 error of 5%, the Sample size is calculated according to the following formula, n= $\:\frac{(\text{Z}1-{\upalpha\:}/2)^2.\text{P}\left(1-\text{P}\right)}{d^2}$ where Z1-α/2 at 5% type 1 error (*p* < 0.05) is 1.96, P is the expected proportion in population based on previous studies and d is the absolute error or precision. Therefore, the sample size n= $\:\frac{\left(1.96\right)^2.\left(0.725\right)(1-0.725)}{\left(0.0794\right)^2}$ =121.5. Based on the formula, the total sample size required for the study is 122.

### Ethical consideration

The research team received ethical approval from the Research Ethics Committee of the Faculty of Nursing at Mansoura University (Ref N 0580), ensuring compliance with World Medical Association’s Code of Ethics (Declaration of Helsinki). Comprehensive explanations about the study’s objectives, methods, expected duration, and potential benefits were provided to the directors of the selected settings. Additionally, the purpose, benefits, and risks of the research study were communicated to the psychiatric and mental health nurses prior to their participation. Informed consent was obtained from each participant after they confirmed their understanding of the study’s details. Participants were assured that their involvement was entirely voluntary and that they could withdraw at any time. To protect participant privacy, all collected data were handled with strict confidentiality, and participants’ anonymity was maintained throughout the study.

### Measures

A variety of self-report measures were used to assess the level of stigma and empathy among psychiatric nurses and to determine the relationships between these variables. Three instruments were utilized: a socio-demographic questionnaire, the Opening Minds Scale for Health Care Providers (OMS-HC), and the Perth Empathy Scale (PES).

A socio-demographic questionnaire was developed by the researchers and included questions such as age, gender, income, work hours, history of mental and physical disability, nationality, religion, marital status, educational level, years of work experience, current area of work, family history of mental illness, participant’s history of mental illness, and previous experience with mental illness patients prior to employment.

Opening Mind Scale for Health Care Providers [32] is a self-report scale that assesses health professionals’ attitudes and behaviors towards people with mental illness, especially the stigma toward mentally ill people. The full OMS-HC contains 20 items with scores that can range from 20 to 100. High scores suggest a more stigmatizing attitude. The scale uses a 5-item Likert scale (strongly disagree = 1, disagree = 2, neither agree nor disagree = 3, agree = 4, and strongly agree = 5). The Opening Mind Scale for Health Care Providers (OMS-HC) does not provide predefined cutoff points for categorizing stigma levels (low or high). Instead, categorization is typically based on the distribution of scores within the study sample. In this study, stigma levels were classified using the median score as a cutoff: Low stigma: Scores below the median. High stigma: Scores equal to or above the median. This method aligns with prior research [33]. which utilized sample-specific thresholds to interpret OMS-HC scores in the absence of standardized cutoffs. Authors of the OMS-HC recommend interpreting scores on a continuous scale, with higher total scores reflecting more stigmatizing attitudes. Thus, the use of a median split is a valid and widely accepted approach to categorize stigma levels. The scale showed good internal consistency, Cronbach’s alpha = 0.82, and satisfactory test-retest reliability, intra-class correlation = 0.66 (95% CI 0.54 to 0.75) [32].

The Perth Empathy Scale [34] is a 20-item self-report measure for clinicians and researchers who want to assess cognitive and affective empathy. The scale uses a 5-point Likert scale, ranging from almost never (1) to almost always (5). Higher scores indicate higher levels of empathy. The original English version of the PES has demonstrated acceptable to good internal consistency reliability, with a Cronbach’s alpha of ≥ 0.70 for its subscales and composite scores.

OMS-HC and PES have been translated into several languages that showed satisfactory reliability and validity. For the present study, the researchers translated the study tools of OMS-HC and PES into Arabic after obtaining permission from the original authors. Five psychiatric and mental health nursing experts tested the translated tools for content validity at the Faculty of Nursing, Mansoura University. Cronbach’s alpha was used to assess the tools’ reliability (0.896 & 0.901, respectively). The Cronbach’s alpha value (internal consistency) of the nurses’ attitude scale is 0.903, and the Perth empathy scale is 0.896.

### Data collection procedure

Data was collected through an online survey introduced on Google Forms over approximately 3 months (from January 2024 to the end of March 2024). The primary investigator collaborated with hospital administrative staff, who distributed the survey link via email and WhatsApp to eligible nurses depending on their preference. The Google Form included a detailed description of the study, informed consent, and the questionnaire. Interested nurses reviewed the study objectives, confidentiality measures, and voluntary nature of participation before proceeding. The survey took approximately 10–15 min. Participation was monitored through periodic follow-ups with administrative staff, and reminder messages were sent via email and WhatsApp to encourage their engagement. All responses were collected anonymously, with no identifying information linked to participants. Data was securely stored in password-protected files accessible only to the primary investigator, ensuring compliance with ethical standards.

Before proceeding with the study, a pilot study was conducted to assess the tools and the study’s feasibility. This pilot study also served as a trial run for the fieldwork, helping the researchers identify potential impediments or issues that might arise during data collection. Additionally, it provided insights into the time required to complete the questionnaire. The pilot study involved 30 participants, including nurses and nursing students, which represented more than 20% of the total study sample. The researchers evaluated the applicability and simplicity of the research techniques and made minimal modifications to the sampling strategy and tools based on the pilot study findings.

### Statistical analysis

Statistical analyses were performed using SPSS for Windows version 20.0 (SPSS, Chicago, IL). Descriptive analysis expressed in mean and standard deviation (SD) was conducted for continuous data, and the calculation of frequency and percentage was used for categorical data. A chi-square test (or Fisher’s exact test when applicable) was used for comparison of variables with categorical data. Pearson’s correlation coefficient was used to determine the correlations between two variables and continuous data. The linear regression analysis model was used to determine the factors that predict empathy toward mentally ill patients among psychiatric and mental health nurses based on socio-demographic factors and stigmatizing attitudes. Additionally, the reliability (internal consistency) test for the questionnaires used in this study was calculated. The statistical significance level was considered at *p* < 0.05.

## Results

**Table 1:** Distribution of the sociodemographic and professional characteristics of the psychiatric nursing (122)

	*n*	%
**Age (Years)**		
< 20	17	13.9
20–30	27	22.1
30–40	78	63.9
**Gender**		
Male	35	28.7
Female	87	71.3
**Monthly Income**		
< 5000	19	15.6
5000–10,000	78	63.9
10,000–15,000	17	13.9
15,000–20,000	8	6.6
**Residence**		
Urban	68	55.7
Rural	54	44.3
**Marital Status**		
Married	86	70.5
Single	36	29.5
**Educational Level**		
Master’s degree (psychiatric nursing)	16	13.1
Doctorate in psychiatric nursing	10	8.2
Diploma	47	38.5
Bachelor’s	49	40.2
**Working hours**		
6–8	20	16.4
8–10	54	44.3
10–12	30	24.6
More than 12	18	14.8
**Experience years**		
< 5	32	26.2
5–10	53	43.4
10–15	29	23.8
> 15	8	6.6

Table 1: This table represents the sociodemographic and professional characteristics of psychiatric nursing. The study sample of 122 participants was primarily aged 30–40 years (63.9%) and mostly female (71.3%). A significant portion had a monthly income between 5000 and 10,000 (63.9%) and lived in urban areas (55.7%). Most were married (70.5%) and held either a bachelor’s (40.2%) or diploma (38.5%) degree. Most worked 8–10 h daily (44.3%) and had 5–10 years of experience (43.4%).

**Table 2:** Distribution of the family medical history of the psychiatric nursing

	*n*	%
**Is there anyone in the family who has a mental illness?**		
No	111	91.0
Yes	11	9.0
**If yes, what is your relation to them?** (*n*** = 11)**		
Uncle / Aunt	5	45.5
Sibling	2	18.2
Husband / Wife	2	18.2
Nephews	2	18.2
**Before you worked in the psychiatric hospital, did you have any previous experience with psychiatric patients?**		
No	88	72.1
Yes	34	27.9
**If yes, was the experience positive or negative?** (*n*** = 34)**		
Negative	14	41.2
Positive	20	58.8
**Have you ever been to a psychiatrist?**		
No	111	91.0
Yes	11	9.0
**Do you suffer from mental problems?**		
No	112	91.8
Yes	10	8.2
**If yes, what do you suffer from?** (*n*** = 10)**		
Depression	6	60.0
Anxiety	5	50.0
OCD	3	30.0
**Do you suffer from organic diseases?**		
No	110	90.2
Yes	12	9.8
**If yes, what do you suffer from?** (*n*** = 12)**		
Chest sensitivity	2	16.7
High blood pressure	7	58.3
Gland inflammation	3	25.0

Table 2: The study sample reveals that most participants (91.0%) have no family history of mental illness, and most (72.1%) had no prior experience with psychiatric patients before working in the psychiatric hospital. Among the few with such experience, the majority found it positive (58.8%). A significant portion of the sample (91.0%) has never visited a psychiatrist, and 91.8% do not suffer from mental problems. Among the 8.2% who do, depression (60.0%) and anxiety (50.0%) are the most common issues. Regarding physical health, 90.2% of participants do not suffer from organic diseases, with high blood pressure being the most prevalent condition (58.3%) among those who do.

**Table 3:** Distribution of psychiatric nurses’ attitude level toward mentally ill patients

	*N*	%
Least stigmatizing (positive attitude)	86	70.5
Most stigmatizing (negative attitude)	36	29.5
Mean ± SD		53.9 ± 8.8

Table 3: The data revealed that 70.5% of nurses exhibited the least stigmatizing attitudes, reflecting a positive attitude. In contrast, 29.5% of the participants demonstrated the most stigmatizing attitudes, indicating a negative attitude. The overall mean attitude score was 53.9 ± 8.8.

**Fig. 1:**
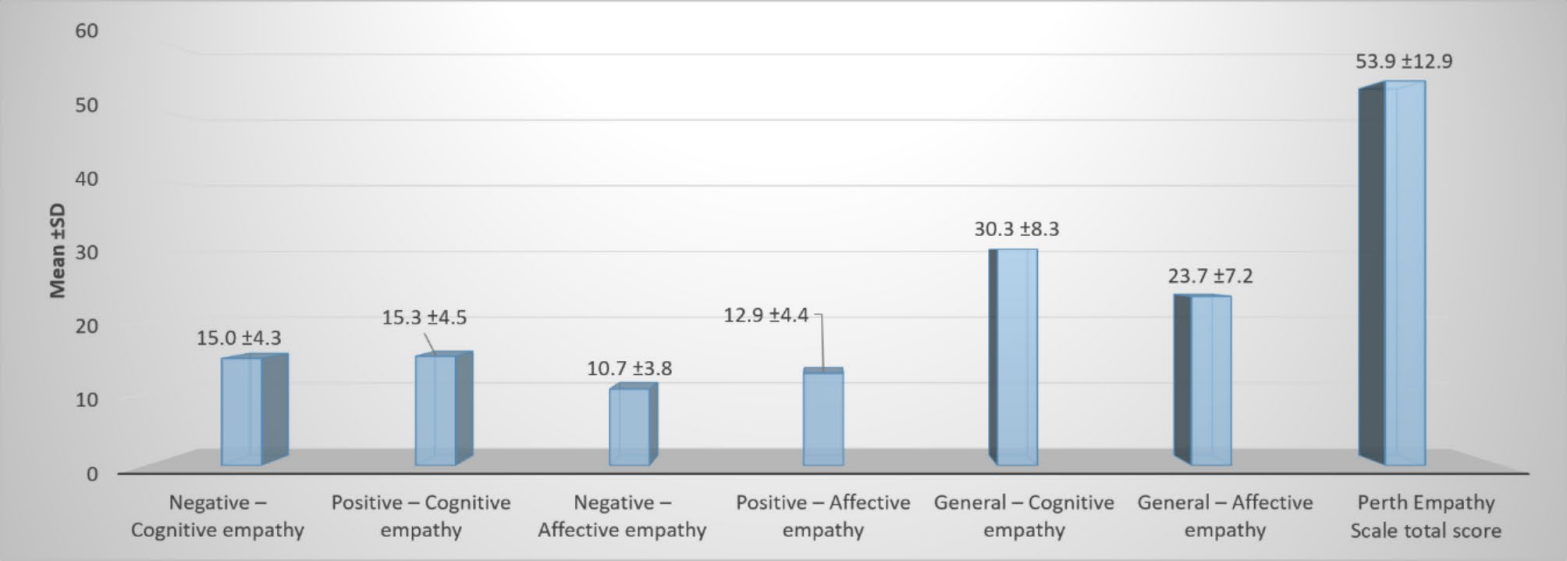
Distribution of the perth empathy scale domains among the psychiatric nurses

The Fig. 1 displays the mean scores and SD for various empathy dimensions measured by the Perth Empathy Scale. General-Cognitive Empathy has the highest mean score at 30.3 (SD ± 8.3), followed by General-Affective Empathy at 23.7 (SD ± 7.2). Positive-Cognitive and Negative-Cognitive Empathy show similar mean scores of 15.3 (SD ± 4.5) and 15.0 (SD ± 4.3), respectively. Positive-affective and Negative-Affective Empathy have lower mean scores, 12.9 (SD ± 4.4) and 10.7 (SD ± 3.8), respectively. The overall Perth Empathy Scale total score is 53.9 (SD ± 12.9).

**Fig. 2:**
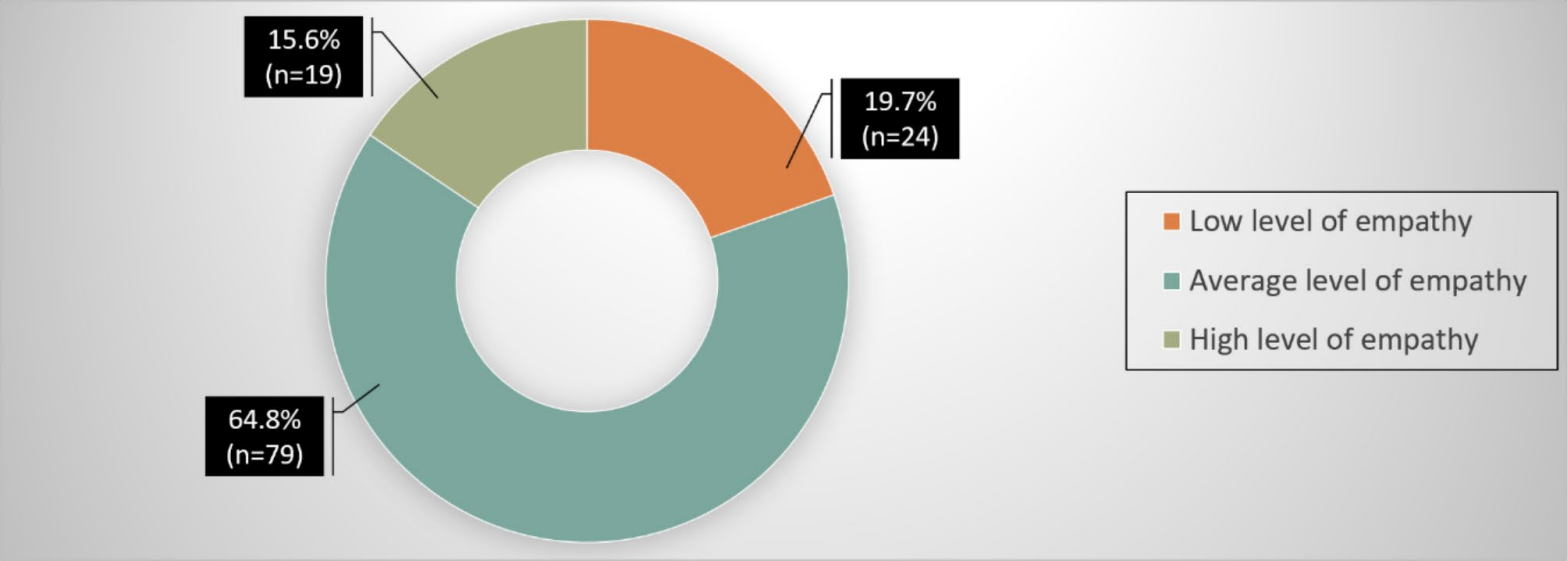
Distribution of perth empathy scale level among psychiatric nurses

The Fig.  2 shows the distribution of empathy levels among the nurses; as measured by the Perth Empathy Scale, the majority, 64.8%, exhibited an average level of empathy. A smaller proportion, 19.7%, demonstrated a low level of empathy. Conversely, 15.6% of the participants showed a high level of empathy.

**Table 4:** Association between the sociodemographic characteristics and attitude level among psychiatric nurses

	Least stigmatizing (*n* = 86)		Most stigmatizing (*n* = 36)		Chi-square / Fisher’s exact test	
	**n**	**%**	**n**	**%**	**X** ^**2**^	***P***
**Age (Years)**						
< 20	9	10.5	8	22.2		
20–30	20	23.3	7	19.4		
30–40	57	66.3	21	58.3	2.935	0.231
**Gender**						
Male	21	24.4	14	38.9		
Female	65	75.6	22	61.1	2.597	0.107
**Monthly Income**						
< 5000	14	16.3	5	13.9		
5000–10,000	58	67.4	20	55.6		
10,000–15,000	10	11.6	7	19.4		
15,000–20,000	4	4.7	4	11.1	3.382	0.336
**Residence**						
Urban	44	51.2	24	66.7		
Rural	42	48.8	12	33.3	2.473	0.116
**Marital Status**						
Married	60	69.8	26	72.2		
Single	26	30.2	10	27.8	0.074	0.786
**Educational Level**						
Master’s degree (psychiatric nursing)	0	0.0	16	44.4		
Doctorate in psychiatric nursing	0	0.0	10	27.8		
Diploma	37	43.0	10	27.8		
Bachelor’s	49	57.0	0	0.0	84.154	< 0.001**
**Working hours**						
6–8	13	15.1	7	19.4		
8–10	38	44.2	16	44.4		
10–12	21	24.4	9	25.0		
More than 12	14	16.3	4	11.1	0.753	0.861
**Experience years**						
< 5	11	12.8	21	58.3		
5–10	38	44.2	15	41.7		
10–15	29	33.7	0	0.0		
> 15	8	9.3	0	0.0	35.593	< 0.001**

Table 4: The table reveals the relationship between sociodemographic characteristics and nursing attitudes toward mentally ill patients. A statistically significant difference was found in educational level and years of experience (*P* < 0.001), with higher education in psychiatric nursing and less experience associated with a more stigmatizing attitude toward psychiatric patients.

**Table 5:** Association between the sociodemographic characteristics and perth empathy level

	Low level of empathy (*n* = 24)		Average level of empathy (*n* = 79)		High level of empathy (*n* = 19)		Chi-square/ Fisher’s exact test	
	**n**	**%**	**n**	**%**	**n**	**%**	**X** ^**2**^	***P***
**Age (Years)**								
< 20	5	20.8	9	11.4	3	15.8		
20–30	3	12.5	19	24.1	5	26.3		
30–40	16	66.7	51	64.6	11	57.9	2.662	0.616
**Gender**								
Male	10	41.7	20	25.3	5	26.3		
Female	14	58.3	59	74.7	14	73.7	2.467	0.291
**Monthly Income**								
< 5000	5	20.8	13	16.5	1	5.3		
5000–10,000	15	62.5	52	65.8	11	57.9		
10,000–15,000	3	12.5	9	11.4	5	26.3		
15,000–20,000	1	4.2	5	6.3	2	10.5	5.087	0.533
**Residence**								
Urban	14	58.3	42	53.2	12	63.2		
Rural	10	41.7	37	46.8	7	36.8	0.702	0.704
**Marital Status**								
Married	14	58.3	58	73.4	14	73.7		
Single	10	41.7	21	26.6	5	26.3	2.124	0.346
**Educational Level**								
Master’s degree (psychiatric nursing)	15	62.5	1	1.3	0	0.0		
Doctorate in psychiatric nursing	7	29.2	3	3.8	0	0.0		
Diploma	2	8.3	37	46.8	8	42.1		
Bachelor’s	0	0.0	38	48.1	11	57.9	91.403	< 0.001**
**Working hours**								
6–8	3	12.5	14	17.7	3	15.8		
8–10	8	33.3	37	46.8	9	47.4		
10–12	9	37.5	17	21.5	4	21.1		
More than 12	4	16.7	11	13.9	3	15.8	3.255	0.776
**Experience years**								
< 5	21	87.5	11	13.9	0	0.0		
5–10	3	12.5	50	63.3	0	0.0		
10–15	0	0.0	18	22.8	11	57.9		
> 15	0	0.0	0	0.0	8	42.1	123.015	< 0.001**

Table 5: The association between sociodemographic characteristics and Perth empathy levels reveals a statistically significant association between Perth empathy levels and educational level, with those holding bachelor’s degrees exhibiting the highest empathy levels compared to other educational backgrounds, as indicated by the chi-square test (χ2 = 91.403, *p* < 0.001). Moreover, individuals with over 15 years of experience demonstrate significantly higher levels of empathy compared to those with fewer years in the field (χ2 = 123.015, *p* < 0.001).

**Fig. 3:**
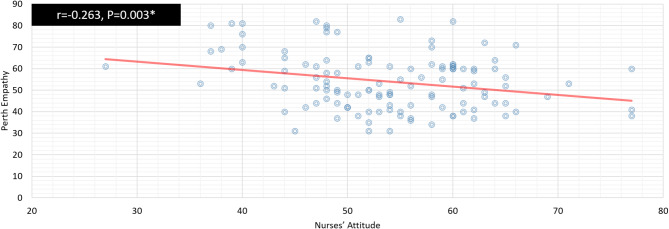
Correlation between nurses’attitude andthe perth empathy score

Figure 3: Reveals a negative correlation between the nurses’ attitude toward mentally ill patients and Perth empathy score as *r* = -0.263, *P* = 0.003*, *r* = − 0.263, *P* = 0.003^*^.

**Table 6:** Linear regression on factors affecting attitude

	Unstandardized Coefficients		Standardized Coefficients	t	Sig.
	B	Std. Error	Beta		
(Constant)	1.365	0.241		5.668	< 0.001**
Educational level	0.126	0.142	0.258	2.619	0.036*
Experience years	0.142	0.148	0.280	2.872	0.023*
Perth empathy	0.150	0.171	0.252	2.565	0.009*

Table 6 displays that educational level (B = 0.126, *p* = 0.036), experience years (B = 0.142, *p* = 0.023), and Perth Empathy (B = 0.150, *p* = 0.009) significantly influence nursing attitudes toward mentally ill patients. Higher education, more years of experience, and greater empathy are associated with more positive attitudes.

**Table 7:** Linear regression on factors affecting perth empathy

	Unstandardized Coefficients		Standardized Coefficients	t	Sig.
	B	Std. Error	Beta		
(Constant)	2.599	0.687		3.781	< 0.001**
Educational level	0.149	0.180	0.285	2.870	0.006*
Experience years	0.197	0.144	0.269	2.611	0.007*
Attitude	0.150	0.171	0.252	2.565	0.009*

Table 7 reveals that educational level (B = 0.149, *p* = 0.006), experience years (B = 0.197, *p* = 0.007), and attitude toward mentally ill patients (B = 0.150, *p* = 0.009) significantly impact Perth Empathy. Higher education, more years of experience, and a positive attitude toward mentally ill patients are linked to higher levels of empathy.

## Discussion

This study was conducted on a sample of psychiatric and mental health nurses to assess stigmatizing attitudes and predictors of empathy toward mentally ill patients. The study found that nearly three quarters of the nurses had a low stigmatizing attitudes toward mental illness (having positive attitudes), while approximately one-third had a high stigmatizing attitudes (having negative attitudes), meaning that most of the participants having positive attitude toward mentally ill patients. Additionally there were no statistically significant differences between male and female nurses regarding their stigmatizing attitude. These findings are consistent with the study by Vagheei et al. 2018 [35], which revealed the total mean of stigma score for psychiatric disorders among students was 57.03 ± 6.27 out of 100, which was a similar score to that found in our study(53.9 ± 8.8). Conversely, our results differ from those of Šimičić et al. 2023 [36], who reported that male nurses exhibited more stigmatizing attitudes toward mentally ill people compared to female nurses. Their study also reported that most nurses perceived mentally ill patients as aggressive and dangerous, and believed that they could not empathize with them. Additionally, other studies have reported negative attitudes among mental health nurses and community health staff. For example, Li et al. 2014 [37]. observed a high level of stigma, negative attitudes, and hesitancy to engage with patients among mental health staff.

In terms of the relationship between sociodemographic factors and stigmatizing attitudes, the current study found a statistically significant association between higher education in psychiatric nursing, years of experience, and a higher stigmatizing attitude toward mental illness. This finding contrasts with the study by Chang et al. 2017 [38] on nursing students in Singapore, which reported that healthcare students held positive attitudes towards help-seeking and individuals with mental illness, despite a preference not to disclose their own mental health conditions. Additionally, our results differ from those of Ebrahimi et al. 2012 [12] who found that bachelor’s and master’s degree students had lower cognitive stigma compared to licensed practical nurses. Our findings also contrast with those of Seman et al. 2024 [39]. which demonstrated a significant correlation between stigma and knowledge about mental illness among nurses. Their study also showed that high levels of knowledge were positively associated with increasing the helping behavior, willingness to communicate about mental health, and reducing blame and anger; these highlight how the education and experience significantly shape positive attitudes among healthcare professionals. The increase in stigmatization attitudes in our study within higher education could be influenced by various factors, including cultural aspects. As noted in Table 1, nearly half of the sample comes from urban areas. This observation aligns with Ran et al. 2021 [40], who reported that the cultural background plays a significant role in shaping the prevalence and manifestation of mental illness stigma.

The present study found a significant relationship between higher stigmatization attitudes and fewer years of experience. In our point of view this could be regarded to lack of clinical skills in dealing with those patients, their poor judgment as new nurses having low self-confidence. This is consistent with Magqadiyane 2020 [41], who reported that nurses with less experience, low management skills, and lower self-confidence are more likely to suffer from anxiety and traumatic experiences in mental health settings. Additionally, the findings align with Giralt Palou et al. 2022 [42], who noted that clinical practice experience was associated with positive changes in attitudes toward mentally ill patients.

Regarding relationship between stigmatizing attitude and empathy, this study found a negative relationship between stigmatizing attitudes and empathy, indicating that a higher stigmatizing attitude is linked to lower empathy scores. When nursing staff often believe that people with mental illnesses are dangerous, unpredictable, and emotionally unstable. They may feel worry, guilt, and hostility toward patients with psychiatric illnesses. Hence empathy which needs nurses to feel and comprehend patients’ emotions will not take place. This explanation supported by Román-Sánchez et al. 2022 [22], who found that high empathy levels were correlated with more positive attitudes towards patients with mental illness and reduced stigma. However, these results contradict Vagheei et al. 2018 [35], who reported that the stigma’s social responsibility score subscale had a negative relationship with empathy, though no significant relationship was found between the stigma total score and other subscales of empathy. Additionally, the findings also differ from Webb et al. 2016 [43], which indicated that empathy and adult attachment did not significantly account for stigma variance.

Regarding empathy among the studied nurses, the results indicated that about two-thirds of the participants had a moderate total empathy score on the Perth Empathy Scale. Specifically, general cognitive empathy was higher than general affective empathy. This finding aligns with several previous studies. For instance, Mousa 2015 [44], found that nursing students at the baccalaureate level demonstrated a high level of empathy after completing their psychiatric course (both theoretical and practical). Similarly, Anandan et al. 2024 [45] reported that psychiatric and mental health nurses had higher mean empathy score (47.71 ± 8.28) towards patients with dual diagnosis, compared to53.9 (SD ± 12.9)in our study. Ebrahimi et al. 2012 [12] also found that the majority of nurses did not feel fear or anger towards persons with mental illness but were more empathetic instead, with nearly all expressing a desire to help mentally ill patients. However our results contrast with the findings of Santos et al. 2023 [46], who studied emotional and cognitive empathy among health-related and non-health-related academic students. They found that affective empathy levels were higher among students in health fields compared to those in exact sciences, while cognitive empathy levels were significantly higher among students in exact sciences compared to those in other fields.

In relation to the main objective of this study, which is examining the predictors of empathy toward mentally ill patients, the study found that a positive attitude toward mentally ill patients is linked to higher levels of empathy. Regarding this result, a little or nearly no study has examined stigma as a predictor of empathy in psychiatric and mental health nurses; however, the opposite is present, as some studies have examined empathy as a predictor of stigma. From those studies, a study by Powell 2014 [47] about stigma against mental illness: the influence of empathy, perspective-taking, exposure to, and familiarity with mental illness revealed that empathy and perspective-taking were not considered the main cause of significant differences in stigma toward mental illness. In our point of view, stigma causes discrimination against and isolation of the patient, which places the person with mental illness in an unfavorable social situation. However, nurses should not become irritable or anxious when patients express negative emotions; instead, nurses should remain empathetic and supportive [45]. That’s why in our study we found a relationship between a decline in stigma and an increase in the level of empathy toward mentally ill patients. Another study by Silke et al. 2017 [48] (conducted adolescents) revealed that empathy was found to have limited effects on adolescents’ explicit and implicit stigmatizing responses.

Finally, from all sociodemographic factors we found that only higher education and more years of experience are the only predictors of a high level of empathy toward mentally ill patients. More specifically, the relation between sociodemographic characteristics and empathy revealed a statistically significant association between Perth empathy levels and educational level, those holding bachelor’s degrees are exhibiting the highest empathy levels compared to other educational backgrounds. Moreover, individuals with over 15 years of experience demonstrate significantly higher levels of empathy compared to those with fewer years in the field. These results align with a study conducted by Román-Sánchez et al. 2022 [22] which revealed that empathy toward patients with mental illness increases with an increase in years of experience. A study conducted on nursing students about predictors of empathy revealed that students who were satisfied and had no intention to leave nursing reported a higher level of empathy, lower self-awareness, and higher levels of perceived stress Hamaideh et al. 2024 [49]. They added also that female nursing students showed a higher score than males on the empathy scale. However the last disagree with our result as in our result there were no statistically significant difference between male and female nurses in predicting empathy with mentally ill patients. It could be regarded to our small sample size. Few studies examined predictors of empathy among psychiatric nurses; however, most studies assessed predictors of stigma toward mentally ill patients. A study conducted by Grover et al. 2020 [50] revealed that from all sociodemographic factors, female gender was the only factor that is correlated with stigma toward mentally ill patients. Also, Ebrahimi et al. 2012 [12] delineated that stigma toward mentally ill patients is correlated with many factors such as educational degree, personal experience of nurses, and interest in the continuation of work in the psychiatric ward.

## Conclusion

The study found that more than two-thirds of the sample exhibited low stigmatizing attitudes toward mentally ill patients. Cognitive empathy was higher than affective empathy among the nurses studied. Factors such as educational level, years of experience, and empathy significantly influenced nursing attitudes toward mentally ill patients. Higher education, more years of experience, and greater empathy were associated with more positive attitudes toward mentally ill patients. Additionally, higher education and more years of experience, along with a positive attitude predicted higher levels of empathy toward mentally ill patients.

### Implications of the study

The findings of this study have significant implications for clinical practice, education, and future research. Targeted interventions, such as workshops, role-playing, and Cognitive Behavioral Therapy (CBT) programs, are recommended to reduce stigma and enhance empathy among psychiatric nurses. Peer reviews should be conducted periodically to assess empathy levels, while public awareness campaigns are essential to promote acceptance of patients with mental illness. Educational programs should integrate empathy-building and stigma-reduction strategies into nursing curricula and professional development. Organizational policies must address burnout through workplace support systems like stress management programs, as burnout negatively impacts empathy, especially in experienced nurses. Future research should explore how stigma and empathy evolve over time and across different nursing career stages, such as specialty training in mental health. Ultimately, fostering empathy can lead to personalized care and improved patient outcomes, highlighting the need to address stigma and empathy to enhance mental health care quality and equity.

### Limitation of the study

The authors faced significant challenges in collecting data from the nurses, as empathy was regarded as a sensitive topic. Considerable time was required to persuade the head nurse, who then worked to reassure the nurses about the confidentiality of their information and encourage their participation in the study. Moreover, many nurses were too occupied with their duties to take part in the study.

## Data Availability

The dataset used during the study are available from the coresponding author on request.
